# Observations on Cataract and Strabismus

**Published:** 1808-04

**Authors:** Richard Carmichael

**Affiliations:** Member of the Royal College of Surgeons in Ireland


*Observations on Cataract and Strabismus.* By Richard Carmichael, Member of the Royal College of Surgeons in Ireland.

AMONG the number of prejudices, which still continue to influence the art of surgery, and retard its improvement, I am inclined to include the practice of delaying to operate for the cataract, till both eyes have been totally deprived of sight.

When we consider the mental sufferings of a patient, who is sensible of the gradual and sure approaches of blindness, we are led to expect the most important reasons against an early operation; but, upon inspecting the different writings upon this part of surgery, no very weighty arguments can, I think, be found, to justify this practice; as a slight view of the subject will convince us: but I can offer a few reasons, not unsupported by facts, which will tend to prove the great advantage, which might result to the patient by the opposite practice of removing the cataract when one eye only is affected.

The unnecessary risque of inflammation, whilst the patient continues to enjoy the sight of one eye, is the chief argument urged against early operation; but, in answer to this objection, I shall merely appeal to those surgeons, who frequently perform the operation by depression; as, from my own experience, I have no hesitation in asserting, that a degree of inflammation, capable of injuring vision, is a very rare consequence of this operation, when properly performed; and it is frequently so slight, as scarely to deserve the name of inflammation. But it must be understood, that I only allude to the operation by depression; for *that* by extraction is by many degrees more difficult to the the surgeon, as well as more hazardous to the patient: and I cannot here avoid observing, that there appears no stronger instance of the influence of fashion, even in matters that interest the health and lives of mankind, than the preference which has been for many years given to the latter operation.

As to the old opinions of the ripeness and unripeness of cataract; the one supposed solid, the other fluid; the one fit, the other unfit for operation; experience has long since proved their erroneousness, and any farther attention to them would be needless, except to remark, that the conclusion drawn from those opinions, handed down from one author to another, is probably one of the causes to which we may attribute the postponement of operating till both eyes are reduced to a state of blindness.

There remains but one more objection against early operation, the only one, indeed, which can be urged with any shew of reason, viz. that the removal of the lens from one eye, while the other continues sound, must cause a difference to the refractive powers of the two eyes, and that double vision must be the consequence. Mr. Samuel Cooper, in his excellent treatise upon Cataract, mentions this objection, but at the same time observes, that he has grounds for believing it to be only a gratuitous supposition, blindly transmittted from one writer to another;" and his opinion is negatively supported by cases to which we are referred, as related by Maitre Jean, and Baron Wenzel, who removed the cataract, in many instances, when only one was affected, but make no mention of double vision having been produced. But a case I shall by and by relate will evince, that double vision is, at first, the consequence of the removal of the cataract when only one eye is affected; but it will also prove, that perfect vision will afterwards spontaneously return. And this circumstance we may attribute either to the power of habit, in causing different points of the retina in both eyes to correspond; or to the inattention of the patient to the weaker impression of light upon the eye which has been deprived of its lens; the latter I think the most probable.

Thus the arguments, which have hitherto prevented surgeons from operating for the cataract, before both eyes become affected, appear, upon examination, to deserve but little consideration: and I shall now advance a few reasons, why the operation should be performed whenever the disease is so fully formed in *one* eye, that we can have no hopes of its removal by medicine.

 The

The effects of sympathy are in no part of the frame so obvious, as in the organs under consideration. Almost every person is sensible, that if an extraneous body falls into one eye, the other shares, in some degree, the inflammation. It is fully known to surgeons, that when cataract is caused by external injury in one eye, the other, upon which no injury was inflicted, frequently becomes affected. In such an instance, we cannot attribute the cataracts to any latent disease of the eyes, or of the constitution; but we must conclude, that the affection of the eye, last attacked, is owing to the consent or sympathetic action which exists between the vessels of both organs.

The origin of cataract, from this cause, was impressed very much upon my mind, by the case of an old man, servant to Mrs. D———, of Granby-row, upon whom I performed the operation by depression, about twelve months since. This man, many years ago, was struck on the eye with the lash of a whip, and a cataract succeeded; the left eye long continued well, but, at length, became diseased, and he was utterly blind for some years previous to the period when I saw him. Both cataracts were operated on by the needle; but it was somewhat extraordinary, (and I shall mention it, though it has no connection with our argument,) that the cataract in the right eye, which had continued upwards of forty years, was fluid; while the other, which had not existed a fourth of that period, was solid, and easily depressed. Thus we find what confidence is to be placed in the former opinions, respecting the degree of ripeness of cataract; which, in place of becoming solid by time, on the contrary, becomes fluid, if this case does not differ from every other. It was as white as milk, and more opaque than any I ever saw.

There is a case related by St. Ives, and noticed since by several authors, which proves, in a most decided manner, the effects of sympathy in the production and dissipation of cataract. A man, having a cataract in his right eye, in consequence of external injury, gradually became blind in the left; but on extracting the cataract of the right eye, that in the left spontaneously disappeared.

Although those cases arose from accident, we may reasonably conclude, that when the disease cannot be ascribed to injury, the same process takes place; for it is a well known fact, that, in such instances, one eye is, in general, diseased for a considerable period before the other becomes affected. This consideration naturally suggests, that, if we remove the disease in the affected eye, it will be be prevented from taking place in the sound one; and the following case is not unfavourable to this conclusion.

Margaret Corcoran, aged 50, had been affected by a cataract of the left eye for upwards of two years; the lens was perfectly opaque; the right eye had been also affected for several months, the lens had lost somewhat its transparency, and she complained of dark spots floating constantly before the eye; a symptom which had also taken place on the commencement of the disease in the other.

On the 9th of July, 1806, I depressed the cataract of the left eye; which was followed by so little inflammation, that, on the 11th it had totally subsided, and she could distinctly perceive minute objects with the eye operated on; but she was desired still to continue her bandage.

On the 21st she came to me, much distressed and terrified at seeing every object double. I found that convex glasses had no effect in removing this imperfection; and that the want of the crystalline lens could not thus be supplied. Besides the inconvenience of double vision, she was also affected by a considerable degree of strabismus, the eye operated upon constantly turning towards the nose, a deformity to which she had never been subject before the operation. I desired her to cover the right eye with a bandage, in order to strengthen the left by exercise. I cannot tell whether she followed my directions, but the squint still continues. However, I had the pleasure of finding, on her return from the country, where she had been for the last six months, that perfect vision had returned; and, although the right eye had not been operated on, nor any remedy applied, that its opacity had disappeared, and both eyes were perfectly transparent.

If I might venture to draw a general inference from this case, I would conclude,

1st. That when one eye is affected by cataract, sympathy may occasion the same complaint in the other; previous to which, its removal from the disordered eye will, probably, prevent its formation in the sound one; and, even if it has commenced, the operation may not only check its further progress, but effect a complete cure.

2dly. That one of the causes of squinting may be, a difference in the refractive powers of the eyes; and, therefore, that the *deformity*, when arising from this cause, is totally incurable; but the *inconvenience*, so far as regards distinctness of vision, may be remedied by means of glasses.

To discover whether any case of strabismus is occasioned by by this cause, it will be only necessary to observe if there is any difference in the distance required for distinct vision, when the patient reads or views any minute object, first with one eye, and then with the other, (the eye not in use being closed); and this simple experiment will also point out the defective eye. Mons. Buffon seems to be of the opinion above insisted on: and I shall relate it, in a quotation from Priestley, as I shall thus have an opportunity of elucidating the subject at once, with the thoughts of those two great philosophers.

"The business of squinting was far more accurately considered by M. Buffon, who, from a great number of observations, found, that the true and original cause of it is, *an inequality in the goodness*, or *in the limits of distinct vision in the two eyes*. When one of the eyes, he says, is much *weaker* than the other, we do not direct it towards the object, but make use of the *stronger* eye only: for the same reason that we commonly make use of the right arm in preference to the left; and, as the image in the *good* eye will be stronger than that in the *weak* one, the impression upon them both will not be so distinct as if the good eye only had been used. It is no wonder, therefore, that such persons chuse to make use of one eye only, and turn the other aside.

"Our author does not assert, that this inequality in the goodness of the eyes is the only cause of squinting; he only says, that it is the general cause, so that others may be termed accidental causes with respect to it. All persons that be examined, if they squinted at all, did so from this cause; and this effect is so necessary, that, he says, it *is not possible to cure a person of squinting, whose eyes differ much in point of goodness, except, perhaps, by first bringing them nearer to an equality, by means of glasses.*

"The reason why those persons who squint, generally turn the weak eye towards the nose, he says is, that in that situation the direction of the axis is as distant as possible from that of the good eye; and besides, in this place, the nose conceals many objects from its view; so that this situation is the least disadvantageous to it of any other.

"M. Buffon concludes this curious memoir with observing, that a weak eye acquires strength by exercise: and, that many persons, whose squinting he had thought to be incurable, on account of the great inequality in the *goodness* of their eyes, having covered their good eye a few minutes only, and, consequently, being obliged to exercise their bad eye for that short time, were themselves surprized surprized at the strength it had acquired; so that measuring their view afterwards, he found it more extended, and judged the squinting to be incurable. In order, therefore, to judge, with any certainty, of the inequality of goodness in the two eyes, and the possibility of the cure of squinting, it is necessary, he says, to cover the good eye for some time, in order to exercise the bad eye, and give it an opportunity of gaining strength; after which, it will be easier to judge, whether the cure be possible or not."—(*Priestley on Vision*, p. 654.)

The language, attributed in the above quotation to M. Buffon, is rather indefinite; and we cannot understand precisely what he means, by applying the terms, goodness and badness, strength and weakness, to the eyes.

It would have rendered his meaning much more evident, if he had described those organs as possessing the refractive power in a greater or lesser degree; but, probably, he overlooked the true cause of this kind of squinting, and supposed it to be occasioned by different degrees of sensibility of the retina. Squinting, attributed to this latter cause, being, probably, curable by exercising the affected eye; while the former kind is evidently beyond the power of removal.

*Cumberland-street, June* 1, 1807.

